# Effects of high-altitude environments on intervertebral disc degeneration and transcriptome profiling of the nucleus pulposus

**DOI:** 10.3389/fcell.2025.1709844

**Published:** 2025-12-12

**Authors:** Jiawei Fu, Rong Tang, Tianfei Ran, Yue Zhou, Bo Huang

**Affiliations:** Department of Orthopedics, Xinqiao Hospital, Army Medical University, Chongqing, China

**Keywords:** intervertebral disc degeneration, high altitude, transcriptome, cell senescence, cell cycle, potential biomarkers

## Abstract

**Background:**

Low back pain (LBP) is a leading cause of disability in elderly individuals, and intervertebral disc degeneration (IDD) is the major contributor to LBP. Aging is an important factor contributing to IDD. Research has indicated that the incidence of lumbar intervertebral disc protrusion is significantly greater in high-altitude areas. However, the effects of a high-altitude environment on IDD and the underlying mechanism remain unclear.

**Methods:**

We identified changes in the intervertebral discs of rats of different ages in a simulated high-altitude, low-pressure, and hypoxic environment. Furthermore, through transcriptome analysis, we investigated genes that are differentially expressed during intervertebral disc aging and degeneration at different altitudes.

**Results:**

Gene Ontology (GO) and Kyoto Encyclopedia of Genes and Genomes (KEGG) analyses revealed that biological processes and cellular senescence are important molecular events and regulatory pathways for the development of IDD in individuals who live in high-altitude environments. In the transcriptome sequencing of rat intervertebral discs with aging and degeneration at different altitudes, the results of the GO and KEGG analyses revealed that cell cycle arrest is a key factor in aging and degeneration. In addition, through overlap analysis, we observed changes in SFN expression in high-altitude environments, and this change may be meaningful.

**Conclusion:**

Our findings clarify the effects of high altitude on disc degeneration and predict potential molecular targets that can be used to identify or intervene in disc degeneration caused by high altitude by mining the common features of NP cells associated with the aging process in HA environments through transcriptome sequencing.

## Introduction

More than 80 million people worldwide live permanently in high-altitude (HA) regions (Tremblay and Ainslie, 2021; Wu et al., 2025). With socioeconomic development, an increasing number of people are entering HA areas for work, tourism and other reasons (Han et al., 2024). Compared with non-high-altitude (NA) regions, HA regions have lower atmospheric oxygen levels, lower air pressure, and intense ultraviolet radiation (Wu et al., 2025). The effects of long-term exposure to HA environments on the human body remain to be fully elucidated. The HA environment accelerates the aging of various tissues and organs (Ni et al., 2025; Kious et al., 2019), leading to an increase in the incidence of degenerative-related diseases (Pang et al., 2025; Chen et al., 2024). Notably, the incidence of lumbar degenerative diseases is significantly greater in individuals living in HA regions (Chen et al., 2024). However, the effects of HA environments on disc degeneration are unclear.

Intervertebral disc degeneration (IDD) is among the leading causes of low back pain (LBP), which is a very common musculoskeletal disease and has resulted in a heavy healthcare burden and high socioeconomic cost globally (Jin et al., 2020; GBD, 2017 Disease and Injury Incidence and Prevalence Collaborators, 2018; Hartvigsen et al., 2018; Knezevic et al., 2021). IDD is a progressive process of structural and functional damage to the intervertebral disc (IVD) characterized by nucleus pulposus (NP) dehydration, annulus fibrosus rupture, endplate calcification, and functional decline under the effects of cellular aging, matrix metabolic imbalance, abnormal mechanical stress, etc., and is influenced by a variety of external factors (Zhao et al., 2025; Zhuang et al., 2024; Bhunia and Mandal, 2019). NP cells are the main functional cells of the IVD. NP cell dysfunction is a central driver of IDD; its ability to synthesize extracellular matrix, such as type II collagen and proteoglycans, is reduced, and matrix-degrading enzymes are abnormally activated (Francisco et al., 2022), leading to an imbalance in the composition of the NP matrix and a reduction in its water content, which can destabilize the mechanical stability of the disc, leading to rupture of the annulus fibrosus, calcification of the endplates, and other degenerative pathological processes (Zehra et al., 2022). It should be noted that the avascular nature of intervertebral discs means that nucleus pulposus cells exist in an oxygen-deprived environment, relying on diffusion from the cartilaginous endplates to obtain oxygen and nutrients (Urban et al., 2004). NP cell senescence is an important event related to aging-associated IDD. Senescent NP cells exhibit irreversible cell cycle arrest, metabolic imbalance and secretory phenotypic abnormalities (Zhang et al., 2020; Zhao et al., 2017). Thus far, research on the mechanism of IDD remains incomplete.

The PI3K/AKT signaling pathway, as a core pathway involved in the regulation of cell survival, metabolism and stress (Yang et al., 2024; Chang et al., 2025), is closely associated with the development of IDD (Liang et al., 2025). The PI3K/AKT signaling pathway is closely related to cell cycle functions (Chang et al., 2025) and has been shown to influence IDD by modulating NP cell senescence (Zhang et al., 2024). However, no study has reported whether it plays a role in the development of IDD among individuals living in HA environments.

In this study, we investigated the impact of high altitude on intervertebral disc degeneration, and through transcriptomics, we revealed genetic expression changes in nucleus pulposus cells during the process of intervertebral disc degeneration under HA conditions. The possible molecular pathways through which the HA environment affects myeloid cells were also analyzed by functional enrichment analysis. We also screened potential molecular targets for IDD intervention, providing a theoretical basis for a deeper understanding of IDD.

## Materials and methods

### Ethics statement

Animal experiments were conducted in accordance with international guidelines for animal research and were approved by the Animal Protection and Ethics Committee of the Army Medical University (approval number: AMUWEC20255372).

### Simulation at different altitudes

The NA (nonhigh-altitude) groups were housed in standard laboratory animal rooms with stable environmental parameters (200–300 m altitude, 97.6–98.8 kPa), where the temperature was maintained at 22 °C ± 2 °C, the relative humidity was 50%–60%, the animals had free access to food and water, and the light intensity was strictly controlled at 200–300 lux. The HA (high-altitude) groups (5800 m altitude, 35.88 kPa) were housed in hypobaric chambers at the Department of High Altitude Operational Medicine, Army Medical University, which simulated HA conditions. Except the altitude parameters, all the other environmental conditions of the HA groups were consistent with those of the plain control groups. To investigate the effects of high altitude on disc degeneration, rats were subjected to simulated HA hypobaric hypoxia at 5800 m for 4 weeks.

### Materials

In this study, 24 specific pathogen-free-grade male Sprague–Dawley (SD) rats, including 12 5-month-old (young) rats (300 ± 45 g) and 12 18-month-old (old) rats (700 ± 75 g), were used as experimental subjects (Novais et al., 2019). All experimental animals were obtained from the Laboratory Animal Center of the Second Affiliated Hospital of Army Medical University [Laboratory Animal Use License No.: SYXK (Yu) 2022–0018]. To minimize the potential impact of baseline imbalances and other confounding factors on the results, we first performed physical examinations on all rats to rule out obvious tail vertebra disorders. We also strictly controlled the body weight of the included SD rats, aiming to reduce the influence of significant individual differences on the study outcomes. Moreover, the animals were randomly assigned to four groups using a random number table: the nonhigh-altitude young group (n = 6), the nonhigh-altitude old group (n = 6), the high-altitude young group (n = 6), and the high-altitude old group (n = 6). Additionally, four subgroups were established: 1. 200–300 m NA 5-month-old rats (NA-Young), 2. 5800 m HA 5-month-old rats (HA-Young), 3. 200–300 m NA 18-month-old rats (NA-Old), and 4. 5800 m HA 18-month-old rats (HA-Old). Aging SD rats with degenerated discs as a positive control (Liebscher et al., 2011), 5-month-old SD rats as the young group, and 18-month-old SD rats as the old group (Novais et al., 2019).

### Magnetic resonance imaging (MRI)

Rats secured in a micro-MRI cradle were anesthetized with isoflurane and evaluated under a magnet (7.0 Tesla, Bruker Biospec 70/20 USR, Bruker BioSpin Corporation, Billerica, MA) using the following scanning parameters: sequence: TURBO RARE; weight-ing: T2; TE: 35 ms; TR: 3000 ms; flip angle: 90; and slice thickness: 0.70 mm. Analytical Software: DICOM Viewer Free (version 5.3). The Pfirrmann grading system is used to assess the degree of intervertebral disc degeneration (grades I to V, with higher grades indicating more pronounced degeneration) (Pfirrmann et al., 2001).

### Histological staining and morphological analysis

Rat caudal intervertebral discs were fixed in 4% paraformaldehyde solution and decalcified for 14 days. The samples were embedded in paraffin, cut into 5-micron-thick sections, and subjected to a variety of staining procedures, including hematoxylin‒eosin (H&E) staining, Safranin O staining (Solarbio, G1371), and Masson staining. H&E staining was used to evaluate the number and morphology of nucleus pulposus (NP) cells. Safranin O staining was applied to assess the proteoglycan content in NP tissue, while Masson staining was employed to observe the morphology of the annulus fibrosus (AF). In healthy intervertebral discs: H&E staining revealed an abundant number of NP cells with regular morphology; Safranin O staining exhibited strong positivity (deep red/orange-red), indicating a high proteoglycan content; Masson staining showed well-organized blue collagen fibers. In degenerative intervertebral discs: H&E staining demonstrated a reduced number of NP cells with abnormal morphology (e.g., spindle-shaped changes); Safranin O staining intensity was significantly weakened (pale red or colorless), suggesting massive proteoglycan loss; Masson staining displayed disorganized blue collagen fibers with tissue fibrosis (He et al., 2022; Ji et al., 2021). Cell counting was performed using ImageJ software.

### Immunohistochemistry (IHC) and immunofluorescence (IF)

Rat caudal intervertebral disc nucleus pulposus tissue were fixed with 4% paraformaldehyde (Biosharp, BL539A) for 24–48 h and decalcified in 10% EDTA (Mengbio, MBB013). OCT (Sakura Finetek, 4583) embedding was performed, and 8 μm-thick frozen sections were prepared. Next, 0.3% Triton X-100 was added, the samples were permeabilized for 15 min, and 5% BSA was added for 30 min; the primary antibody was added dropwise (see Table 1 for details of the antibody used and the concentration of the dilution), and the samples were incubated at 4 °C overnight, after being washed, the samples were incubated with corresponding secondary antibodies for 1 h at 37 °C. The IHC sections for immunoactivity were visualized with a DAB Substrate Kit (ZSGB-BIO, ZLI-9018), and counterstained with hematoxylin (Solarbio, G1080). The sections for IF staining were mounted with Antifade Mounting Medium (Beyotime, P0131) before being captured under an inverted microscope (Nikon Ts2-FL, Japan), and a fluorescence microscope (Zeiss LSM880), respectively. and the average fluorescence intensity was quantified by ImageJ software.The antibodies used in the experiments are shown in Table 1. Chondroitin Sulfate (CS) is used to reflect the GAG content in intervertebral discs, quantifying GAG levels as a standard for assessing disc degeneration (Beckstein et al., 2008; Bezci et al., 2019; Johnston et al., 2022; Winkler et al., 2014).

**TABLE 1:** Primary antibodies.

Primary antibody	Manufacturer	Catalog number
ACAN	PTG	13880-1-AP
COL2A1	Arigobio	ARG20787
MMP3	Abways	CY5188
SFN(14-3-3σ)	Aladdin	Ab086611
CS	Abcam	Ab11570
COL1A1	Santa	SC-52658
P16	Invitrogen	MA5-17142

### Collection of rat caudal intervertebral disc nucleus pulposus tissue

First, the rats were anesthetized with 0.2% sodium pentobarbital (0.3 mL/100 g) and sacrificed by cervical dissection. Next, the tail was routinely disinfected, and the skin and subcutaneous tissue were incised along the posterior midline. Muscles, tendons, and ligaments were bluntly separated with hemostatic forceps to expose the intervertebral disc. Finally, an incision is made at the annulus fibrosus to access the milky white nucleus pulposus tissue.

### RNA sequencing

Total RNA was isolated from the specified biological materials using TRIzol reagent (Thermo Fisher, cat. no. 15596026) following the manufacturer’s recommended protocol. After RNA extraction, the samples were treated with DNase I (NEB, cat. no. M0303L) to prevent DNA contamination. The purity of the extracted RNA was assessed by measuring the A260/A280 ratio using a Nanodrop OneC spectrophotometer (Thermo Fisher). The integrity of the RNA was verified using the LabChip GX Touch system (Revvity). The concentration of the qualified RNA was then determined using a Qubit 3.0 fluorometer with a QubitTM RNA Broad Range Assay kit (Thermo Fisher, cat. no. Q10210).

For library preparation, total RNA was used as input for RNA sequencing library preparation utilizing the KCTM Digital mRNA Library Prep Kit (Seqhealth Tech. Co., Ltd., Wuhan, China) according to the manufacturer’s instructions. The kit eliminates duplication bias and errors in the PCR and sequencing steps by using a unique molecular identifier (UID) of 12 random bases to label the cDNA molecules. The library preparation included the enrichment of PCR products corresponding to fragments ranging from 200 to 500 base pairs. The enriched libraries were quantified and sequenced on a Novaseq X Plus platform (Illumina) using a PE150 sequencing model to generate paired-end reads. Bioinformatics analysis was performed based on three independent sequencing datasets.

### Identification and analysis of differentially expressed genes (DEGs)

DEGs and differentially expressed transcripts between groups were identified using the edgeR package (version 3.40.2), and a p value < 0.05(The p value was not FDR-adjusted and represents the result of the original statistical test.) and a fold change >2 were used.

### Gene Ontology (GO) and Kyoto Encyclopedia of Genes and Genomes (KEGG) enrichment analyses

GO and KEGG analyses of the DEGs were performed using KOBAS software (version 2.1.1) with a p value cutoff of 0.05 to determine statistically significant enrichment.

### Identification of potential biomarkers

To identify biomarkers for predicting HA-associated intervertebral disc degenerative diseases, we performed overlap analyses of covariable genes in rats of different ages at high altitude with covariable genes during the aging process of rats at different altitudes and overlapped the covariable genes obtained from the abovementioned genes with the cell cycle-associated genes (https://www.genome.jp/kegg/pathway) again.

### Statistical analysis

Reproducible experiments including cell experiments and staining experiments were performed independently at least three times, while bioinformatics analyses were completed based on data from three independent sequencing runs. Statistical analysis was performed using Prism 9 (GraphPad, La Jolla, CA, United States) with data presented as whisker box plots showing all data points with median and interquartile range and maximum and minimum values. Pfirrmann grading employs the Kruskal–Wallis H test with Dunn’s *post hoc* test (Bonferroni correction). Differences between distributions were checked for normality using Shapiro-Wilk tests. For multiple groups comparison, one-way ANOVA was used for normally distributed data, whereas non-normally distributed data was analyzed using Kruskal–Wallis test followed by Dunn’s multiple comparison test.

## Results

### IVD in rats of different ages exhibit varying degrees of degeneration under high-altitude conditions

**FIGURE 1:**
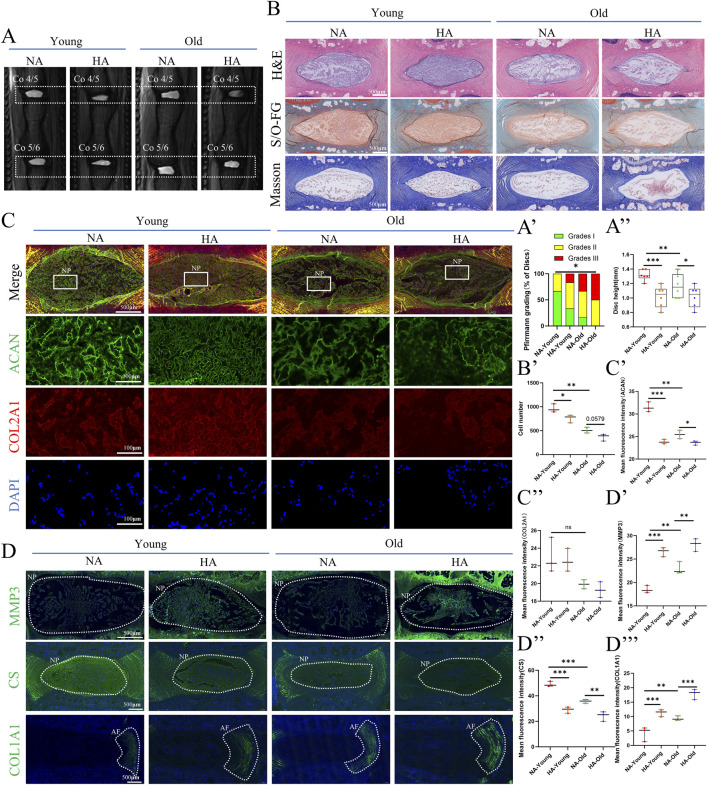
HA causes IDD in rats of different ages. **(A)** Representative MR images of IVDs from rats in different groups (young = 5 months old, old = 18 months old, HA = high altitude<5800 M>, NA = nonhigh altitude<200 M>). **(A’,A”)** Pfirrmann grading and intervertebral disc height (n = 3; 2 IVDs were detected in each rat, and a total of 6 IVDs were counted). **(B)** H&E, safranin O and Masson staining of rat caudal IVD sections from different groups. **(B’)** Number of Nucleus Pulposus Cells (n = 3). **(C,C’,C”)** Immunofluorescence staining of ACAN and collagen II in caudal IVD sections from different groups. Measurement of mean fluorescence intensity (n = 3). **(D,D’,D”,D”’)** Immunofluorescence staining of MMP3, CS and COL1A1 in caudal IVD sections from different groups. Measurement of mean fluorescence intensity (n = 3). * = p < 0.05, ** = p < 0.01, *** = p < 0.001, ns = no statistical significance. Abbreviations: COL2A1: collagen II; ACAN: Aggrecan; MMP3: Matrix Metalloproteinase - 3; CS: chondroitin sulfate; COL1A1: Collagen Type I Alpha 1 Chain.

In the young rats, disc height was significantly lower in the HA group (Figures 1A,A’,A”). H&E, safranin O and Masson staining of paraffin-embedded sections of rat IVD specimens revealed significant degenerative changes at high altitude, Including reduced cell numbers, decreased matrix staining intensity, and disorganized annular fiber arrangement. It should be noted that morphological changes were more pronounced in the old group and appeared insignificant in the young group. (Figures 1B,B’). Immunofluorescence staining revealed that the HA environment reduced the expression levels of aggrecan (ACAN) in NP cells, but collagen type II (COL2A1) showed no significant change in NP cells. (Figures 1C,C’,C”), Changes in ACAN suggesting impaired synthesis of the extracellular matrix. Compared with the NA environment, the HA environment resulted in higher levels of matrix metalloproteinase 3 (MMP 3) expression, suggesting abnormal extracellular matrix catabolism in the NP. The decrease in CS indirectly indicates a reduction in GAG content within the nucleus pulposus tissue at high altitudes, while the elevation of COL1A1 suggests increased fibrosis in the annulus fibrosus of the intervertebral disc (Figures 1D,D’,D”,D’”). Based on a comprehensive evaluation of the aforementioned imaging, histological, and molecular biological findings, SD rats exhibited a trend toward degenerative changes at 5 months of age in the high-altitude environment, with significant alterations in degenerative markers. However, by 18 months of age, the degenerative changes in SD rats under high-altitude conditions became markedly more pronounced.

### Differential gene expression analysis of NP tissues

On the basis of transcriptome sequencing, the influence of high altitude on the expression of the NP gene was further clarified. The amount of clean data from each sample was greater than 6.59 Gb, and the sequence alignment rate with the reference genome was greater than 96.44%. Therefore, our RNA sequence data effectively reflected the gene expression changes in the sample genome.

**FIGURE 2:**
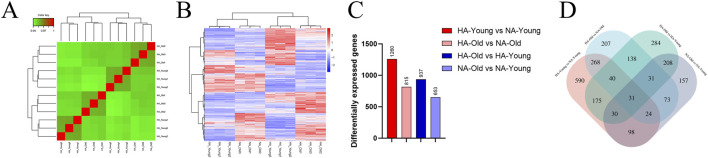
DEGs in the intervertebral discs of rats at different altitudes and of different ages. **(A)** Biological repeat correlation assay for gene expression level samples. **(B)** Heatmap of differential gene clustering. **(C)** Number of DEGs in different comparison groups. **(D)** Comparative analysis of DEGs.

The biological repeatability correlation test for gene expression level samples revealed correlations among the samples (Figure 2A). Under the criteria of a p value < 0.05 and a fold change >2, an overall hierarchical clustering plot of all the DEGs in all the comparison groups clustered by expression, with red indicating highly expressed genes and blue indicating genes with low expression (Figure 2B). A total of 1260 DEGs were identified between the NA-Young and HA-Young groups, 815 DEGs were identified between the NA-Old and HA-Old groups, 937 DEGs were identified between the NA-Old and HA-Young groups, and 653 DEGs were identified between the NA-Old and NA-Young groups (Figure 2C). A Venn diagram shows the overlapping DEGs among the four groups (Figure 2D). A heatmap and volcano map showing the DEGs in the different comparison groups are shown in Supplementary Figure S1.

### GO function and KEGG pathway analysis of the DEGs

**FIGURE 3:**
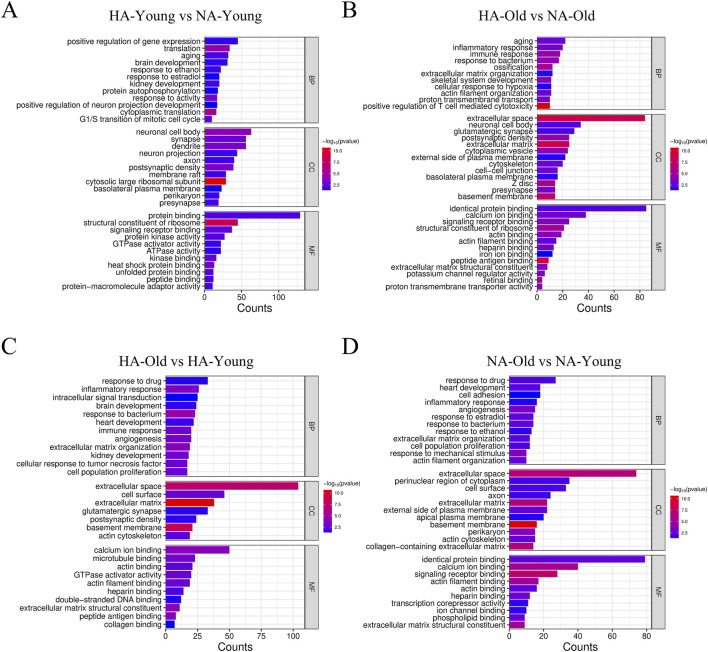
GO enrichment analysis of DEGs. **(A)** HA-YOUNG vs. NA-YOUNG. **(B)** HA-OLD vs. NA-OLD. **(C)** HA-OLD vs. HA-YOUNG. **(D)** NA-OLD vs. NA-YOUNG. HA-YOUNG = 5-month-old rats at high altitude (5800 m); NA-YOUNG = 5-month-old rats at nonhigh altitude (200 m); HA-Old = 18-month-old rats at high altitude (5800 m); NA-Old = 5-month-old rats at high altitude (200 m). BP: biological process; CC: cellular component; MF: molecular function.

GO enrichment analysis was performed to analyze the biological functions of the DEGs. After 4 weeks of high-altitude exposure, the biological processes (BP) enriched by differentially expressed genes (DEGs) in 5-month-old rats included positive regulation of gene expression, translation, aging, and G1/S transition of the mitotic cell cycle. The enriched cellular components (CC) encompassed neuronal cell body, dendrite, and synapse, while the molecular functions (MF) included protein binding, structural constituent of ribosome, and signaling receptor binding (Figure 3A). In contrast, for 18-month-old rats subjected to the same high-altitude treatment, the enriched BP of DEGs included aging, inflammatory response, immune response, and cellular response to hypoxia; the enriched CC included extracellular space, neuronal cell body, and glutamatergic synapse; and the enriched MF included identical protein binding, calcium ion binding, and signaling receptor binding (Figure 3B). Young rats (5-month-old) primarily exhibit abnormalities in gene expression and cell cycle regulation, whereas old rats (18-month-old) are characterized by enhanced aging, inflammatory, and immune responses.

Under high-altitude conditions, the common BP enriched by DEGs between 18-month-old and 5-month-old rats included response to drug, inflammatory response, intracellular signal transduction, and cell population proliferation; the shared CC included extracellular space, cell surface, extracellular matrix, and collagen-containing extracellular matrix; and the overlapping MF included calcium ion binding, microtubule binding, and actin binding (Figure 3C). In the non-high-altitude environment, the BP enriched by DEGs between 18-month-old and 5-month-old rats included response to drug, heart development, inflammatory response, and cell population proliferation; the CC included extracellular space, cell surface, and extracellular matrix; and the MF included identical protein binding and calcium ion binding (Figure 3D). The molecular changes in the aging process at high altitudes involve overlapping phenomena such as disrupted intracellular signaling, cytoskeletal dysfunction, and damage to the collagen matrix. In non-high-altitude environments, these differences are primarily associated with normal physiological aging.

**FIGURE 4:**
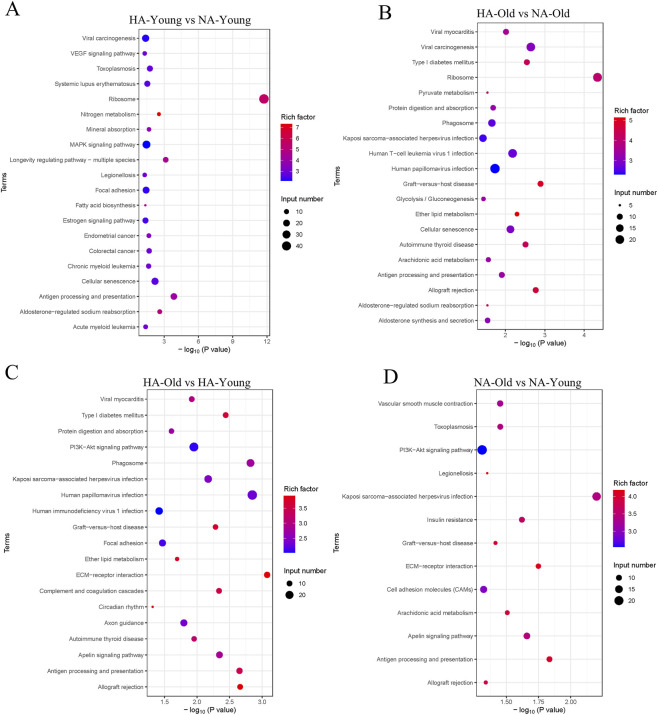
KEGG pathway analysis of DEGs. **(A)** HA-YOUNG vs. NA-YOUNG. **(B)** HA-OLD vs. NA-OLD. **(C)** HA-OLD vs. HA-YOUNG. **(D)** NA-OLD vs. NA-YOUNG. HA-YOUNG = 5-month-old rats at high altitude (5800 m); NA-YOUNG = 5-month-old rats at nonhigh altitude (200 m); HA-Old = 18-month-old rats at high altitude (5800 m); NA-Old = 5-month-old rats at high altitude (200 m).

KEGG enrichment analysis showed that after 4 weeks of high-altitude exposure, the differentially expressed genes (DEGs) in 5-month-old rats were enriched in pathways including Ribosome, Longevity regulating pathway, and Cellular senescence (Figure 4A). For 18-month-old rats, their DEGs after 4 weeks of high-altitude exposure were enriched in pathways such as Ribosome, Cellular senescence, and Glycolysis/Gluconeogenesis (Figure 4B). These results indicate that after 4 weeks of high-altitude exposure, DEGs in both 5-month-old and 18-month-old rats are commonly enriched in the Ribosome and Cellular senescence pathways, with the presence of age-specific enriched pathways. Under high-altitude conditions, the differentially expressed genes (DEGs) between rats of different ages were enriched in pathways including ECM-receptor interaction, PI3K-Akt signaling pathway, and Axon guidance (Figure 4C). In contrast, under non-high-altitude conditions, the DEGs between rats of different ages were mainly enriched in pathways such as ECM-receptor interaction, Apelin signaling pathway, and PI3K-Akt signaling pathway (Figure 4D). These results demonstrate that regardless of high-altitude or non-high-altitude environments, the DEGs between rats of different ages are consistently enriched in the ECM-receptor interaction and PI3K-Akt signaling pathways, while there are differences in environment-specific enriched pathways.

### Functional analysis of common DEGs between young and old age groups at different altitudes

**FIGURE 5:**
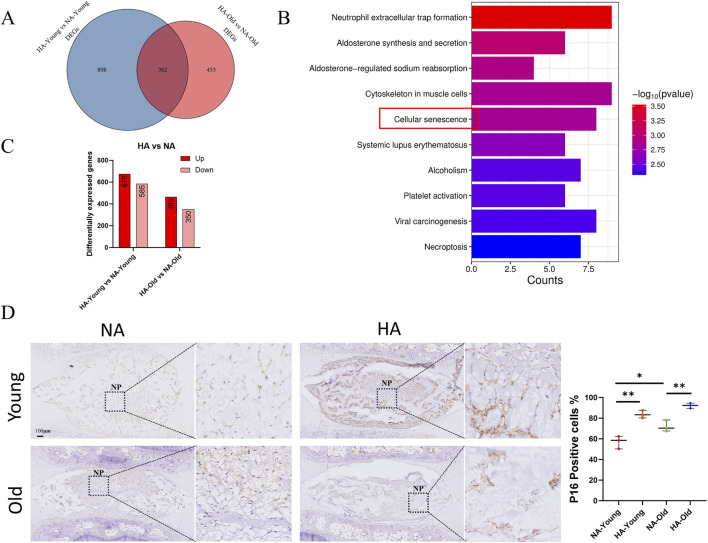
Common underlying mechanism of IDD in rats of different ages due to high altitude. **(A)** Shared DEGs in HA-YOUNG vs. NA-YOUNG and HA-OLD vs. NA-OLD. **(B)** KEGG pathway analysis of shared DEGs. **(C)** Number of upregulated and downregulated genes. **(D)** Immunohistochemistry of P16 in caudal IVD sections from different groups. Measurement of positive cell rate (n = 3). * = p < 0.05, ** = p < 0.01, *** = p < 0.001, ns = no statistical significance.

To elucidate the general pattern of IDD caused by high altitude, we further analyzed the genes whose expression differed between the HA-Young vs. NA-Young and HA-Old vs. NA-Old groups. The results revealed 362 DEGs (Figure 5A). These genes were defined as “HA-related genes.” KEGG enrichment analysis showed that these 362 genes were associated with pathways including cellular senescence and necroptosis (Figure 5B). As previously indicated (Figures 4A,B), after 4 weeks of high-altitude exposure, the differentially expressed genes in both 5-month-old and 18-month-old rats were enriched in the Ribosome and Cellular senescence pathways. Based on the KEGG enrichment analysis of “HA-related genes”, we focused more on the research of the Cellular senescence pathway. The HA-young vs. NA-young DEGs included 675 relatively upregulated genes and 585 relatively downregulated genes in the HA-young group, and the HA-old vs. NA-old DEGs included 465 relatively upregulated genes and 350 relatively downregulated genes in the HA-old group (Figure 5C). Notably, under high-altitude conditions, the expression level of P16 protein in the nucleus pulposus tissue of the caudal intervertebral disc was increased in both 5-month-old and 18-month-old rats, indicating that high-altitude environment exacerbates the senescence of nucleus pulposus cells (Figure 5D). GSEA revealed that in the four comparison groups, the expression of genes related to cell aging bioprocesses (Supplementary Figure S2A), cellular senescence bioprocesses (Supplementary Figure S2B), and cell cycle signaling pathways (Supplementary Figure S2C) decreased with increasing altitude and age. GSEA results indicate that biological processes including cell aging, cellular senescence, and cell cycle are activated to some extent under high-altitude environmental stress and during the aging process, though this activation is not significant.

For each of the above upregulated genes and downregulated genes, 202 genes were coupregulated (Supplementary Figure S3A), implying that those 202 genes were coregulated at high altitude in the young and old age groups. KEGG enrichment analysis revealed that these 202 genes were associated with cellular senescence, the AGE-RAGE signaling pathway, etc. (Supplementary Figure S3B), and there were 143 codownregulated genes (Supplementary Figure S3C), implying that these 143 genes were coregulated at high altitude in the young and old age groups. KEGG enrichment analysis revealed that these 143 genes were associated with AMPK signaling, glycolysis/gluconeogenesis, and the PPAR signaling pathway (Supplementary Figure S3D).

### Functional analysis of common DEGs involved in senescence processes between the HA and NA groups

**FIGURE 6:**
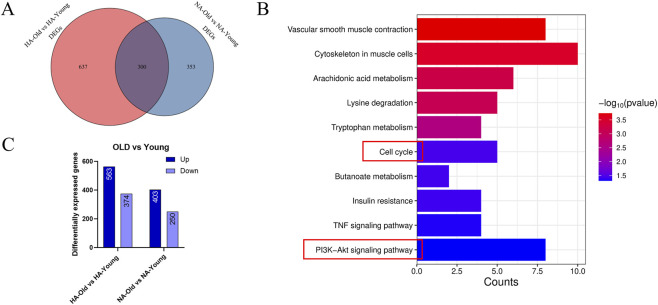
Common underlying mechanisms of age-related degeneration of intervertebral discs in rats at different altitudes. **(A)** Shared DEGs in HA-OLD vs. HA-YOUNG and NA-OLD vs. NA-YOUNG. **(B)** KEGG pathway analysis of shared DEGs. **(C)** Number of upregulated and downregulated genes.

To elucidate the common features of senescence processes between the HA and NA groups, we further performed in-depth analyses of the DEGs between HA-Old and HA-Young and between NA-Old and NA-Young. HA-Old vs. HA-Young and NA-Old vs. NA-Young had 300 common DEGs (Figure 6A), and those genes were defined as “aging-related genes.” KEGG enrichment analysis demonstrated that those 300 genes were associated with the PI3K-Akt signaling pathway, cell cycle, and TNF signaling pathway (Figure 6B). The HA-Old vs. HA-Young DEGs included 563 relatively upregulated genes and 374 relatively downregulated genes in the HA-Old group, and the NA-Old vs. NA-Young DEGs included 403 relatively upregulated genes and 250 relatively downregulated genes in the NA-Old group (Figure 6C). For each of the above upregulated genes and downregulated genes, 179 genes were coupregulated (Supplementary Figure S4A), implying that these 179 genes were coregulated at high altitude in the young and old age groups. The results of the KEGG enrichment analysis revealed that these 179 genes were associated with the Ras signaling pathway, TNF signaling pathway, etc. (Supplementary Figure S4B). A total of 112 genes were codownregulated (Supplementary Figure S4C), suggesting that these 112 genes were coregulated at high altitude in the young and old age groups. KEGG enrichment analysis revealed that these 112 genes were enriched in the TNF signaling pathway, mTOR signaling pathway, and ECM‒receptor interaction pathway (Supplementary Figure S4D).

### Molecular changes in high-altitude environments

**FIGURE 7:**
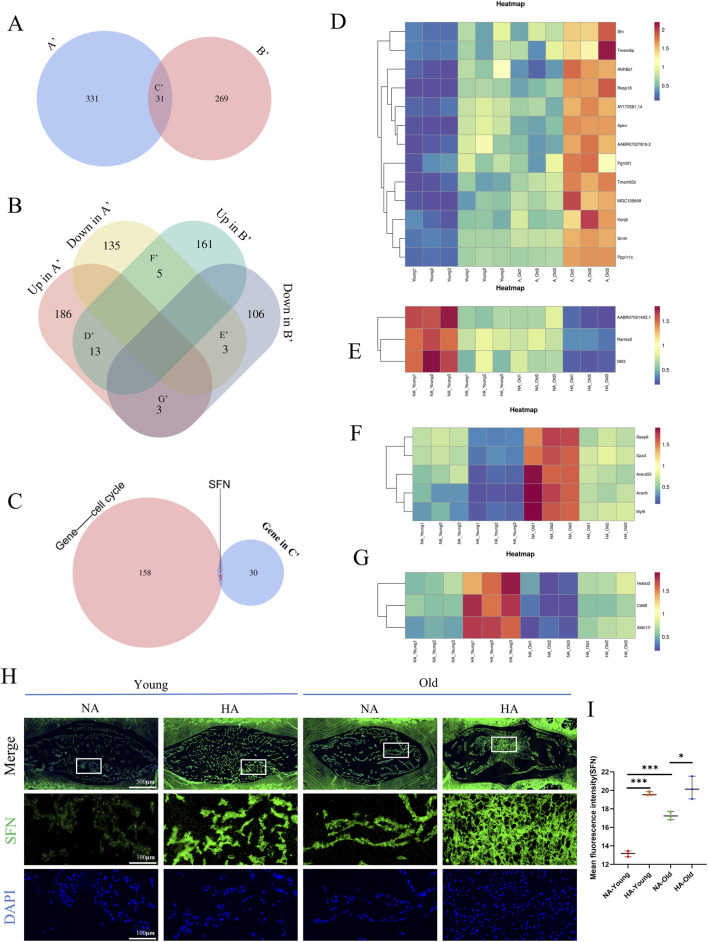
Common potential biomarkers for HA-associated IDD and age-associated IDD. **(A)** Shared DEGs (A’: Shared DEGs in HA-YOUNG vs. NA-YOUNG and HA-OLD vs. NA-OLD; B’: Shared DEGs in HA-OLD vs. HA-YOUNG and NA-OLD vs. NA-YOUNG). **(B)** Comparative analysis of up- and downregulated genes (Up in A’: upregulated in A’; Down in A’: downregulated in A’; Up in B’: upregulated in A’; Down in A’: downregulated in A’). **(C)** Overlap of gene in C’ with cell cycle related genes. **(D–G)** Differentially Expressed Gene Heatmap. **(H,I)** Immunofluorescence staining of SFN in caudal IVD sections from different groups. Measurement of mean fluorescence intensity (n = 3). * = p < 0.05, ** = p < 0.01, *** = p < 0.001, ns = no statistical significance.

To identify meaningful molecular targets at the gene level, we reanalyzed the DEGs described above. Comparative analysis of “altitude-related genes” and “aging-related genes” revealed 31 common DEGs (Figure 7A), 13 of which were jointly upregulated, three of which were jointly downregulated, and 8 of which had opposite expression changes (Figure 7B). A heatmap based on the expression levels of those genes was constructed (Figures 7D–G).

Combined with the results of the analyses described in the previous section, we compared those 31 covariant genes with genes related to the cell cycle signaling pathway, and the results revealed that SFN (14-3-3σ) was the only gene involved (Figure 7C). Immunofluorescence staining of NP tissue sections revealed that SFN was upregulated not only during aging but also at high altitudes (Figures 7H,I). These results suggest that high altitude affects the expression of SFN. The altered expression of SFN may be a key factor in intervertebral disc degeneration at high altitudes.

## Discussion

High altitudes are challenging environments for humans because of the combination of harsh conditions, including low air pressure and low oxygen levels, placing extreme demands on human activities and ecosystems (Bai et al., 2025; Zidan et al., 2025; Chakraborty and Roychoudhury, 2022). Nevertheless, high altitude is part of nature, and it is necessary to fully recognize and understand the effects of high altitude on human health. HA environments have been reported to accelerate aging and contribute to the development of diseases associated with aging (Wu et al., 2025). Several aging-related changes in HA environments, such as telomere shortening, mitochondrial dysfunction and metabolite accumulation, trigger degenerative pathologies (Pang et al., 2025; Gao et al., 2023; Gonzales et al., 2022).

IDD is the most common cause of low back pain and a classic degenerative disease (Ogaili et al., 2025; Yang et al., 2025), and aging is among the most central drivers of disc degeneration (Novais et al., 2019; Song et al., 2024; Samanta et al., 2023). Chen et al. (2024) reported a significant increase in the number of patients with lumbar disc herniation with increasing altitude. However, their study did not clarify the effects of HA on disc degeneration. In this study, the degeneration of the caudal intervertebral disc was investigated for the first time by simulating an HA environment under low-pressure, low-oxygen conditions and using an aging degeneration model as a positive control group. Rat and mouse aging caudal disc models are widely recognized as classic models for studying IDD (Holguin et al., 2014; Gruber et al., 2007), and we used these models as control models in this study. Notably, the low-altitude model of aging is well established and is commonly used in related studies (Novais et al., 2019), whereas the HA model of aging is poorly researched; thus, we also studied the changes in the intervertebral disc in aging rats at high altitude.

The avascular nature of the intervertebral disc dictates that nucleus pulposus cells survive in a hypoxic environment. Under high-altitude conditions, systemic hypoxia leads to a decrease in blood oxygen partial pressure (PaO_2_), narrowing the oxygen partial pressure gradient across the endplates and directly reducing the driving force for oxygen diffusion (Risbud et al., 2010; Forrer et al., 2023). High altitude-associated polycythemia may increase blood viscosity, indirectly impairing microcirculatory perfusion around the endplates and further compromising oxygen diffusion efficiency (Dong et al., 2020; Li et al., 2015). This may represent a link through which systemic hypoxia impacts the intervertebral disc microenvironment (Bartels et al., 1998). The pathological feature of endplate calcification during intervertebral disc degeneration further exacerbates the survival environment of degenerated discs in high-altitude settings, which is consistent with our research. Our results demonstrate that rats in the old group exhibited more significant degeneration under high-altitude conditions, whereas those in the young group appeared to possess a certain degree of adaptability. Our results indicate that intervertebral disc degeneration occurs to a certain extent under high-altitude conditions. In the Young group, this degeneration is mainly characterized by molecular changes, while in the old group, phenotypic changes appear more prominent, accompanied by significant molecular biological alterations. This may be attributed to the physiological structure of the intervertebral disc. Specifically, the aging process in rats is often associated with intervertebral disc degeneration, which impairs the nutrient transport capacity of the endplates. This renders nucleus pulposus cells more sensitive to systemic hypoxia, leading to a faster degeneration rate of the intervertebral disc in high-altitude environments. In conclusion, we propose that the high-altitude environment induces a degenerative trend in the intervertebral disc.

More interestingly, the significantly enriched GO and KEGG pathways in NP cells from rats of different ages at the transcriptional level in the HA environment indicated senescence. Cellular senescence is important for NP dysfunction, leading to intervertebral disc degeneration (Silwal et al., 2023; Wu et al., 2022), which suggests that a high-altitude environment may further contribute to NP cellular senescence and accelerate IDD. Given the unique characteristics of HA environments, the features of aging in HA environments may differ from those in low-altitude environments; thus, analyzing the common features of the aging process at different altitudes is meaningful. We found two genes that were associated with both cell proliferation and the PI3K/AKT signaling pathway through enrichment analysis of genes related to the aging process at different altitudes. One of the characteristics of cellular senescence is irreversible cell cycle disruption at the G1/S phase, and the result of this disruption is a decrease in cell proliferation (He et al., 2023; Liu et al., 2024). Activation of the PI3K/Akt pathway accelerates the cell cycle by upregulating cyclin D1, inhibiting cyclin D1, inhibiting p21 and other cycle-regulating molecules and promoting the transition from the G1 phase to the S phase (Liang and Slingerland, 2003). It can also activate mTOR and other downstream signals to promote protein synthesis and inhibit apoptosis, thus enhancing cell proliferation (Vadlakonda et al., 2013; Hu et al., 2004). These findings suggest that the HA environment accelerates disc degeneration by affecting NP senescence and that alterations in the PI3K/AKT pathway and cell proliferation are key pathways leading to the aging of rats at both high and low altitudes. Changes in the expression of the senescence marker P16, the PI3K/AKT signaling pathway marker pAKT, and the cell proliferation marker P53 in the intervertebral discs of rats in HA environments and aging rats increase the reliability of the above inference, but more studies are needed to confirm these findings.

SFN (stratifin, 14-3-3σ) is a member of the 14-3-3 family of proteins and is located mainly in the cytoplasm, where it functions in intracellular shuttling. As a scaffolding protein, it regulates the cell cycle, DNA damage repair and apoptosis by binding to target proteins and often acts as a tumor suppressor to inhibit abnormal proliferation (Chen et al., 2017; Mohammad and Yaffe, 2009; Winter et al., 2021). The relevant regulatory pathways include the p53 pathway (whose expression can be induced by p53) (Yang et al., 2003; Hermeking et al., 1997), and they cross-interact with PI3K/Akt, MAPK and other pathways and function through the regulation of Cyclin B1 and other molecules (Song et al., 2021; Yang et al., 2006; Shao et al., 2016; Zhou et al., 2018). Our results demonstrate that SFN is significantly upregulated under both high-altitude and aging conditions. Furthermore, GO and KEGG enrichment analyses reveal that high-altitude environments induce IDD primarily through the cellular senescence pathway, whereas the PI3K/Akt and cell cycle pathways are identified as key molecular cascades mediating the aging process. Integrating the established molecular functions of SFN with existing literature, we reasonably hypothesize that SFN serves as a critical responsive molecule to high-altitude stress and aging, participates in the regulation of IDD progression, and thus represents a potential therapeutic target for alleviating or treating IDD. In future work, we will further validate the effects of altered SFN expression levels on intervertebral disc tissue and nucleus pulposus cells through *in vivo* and *in vitro* experimental models.

Our study has some limitations: Without baseline measurements, individual differences were excluded solely through experimental randomization and physical examination. However, based on prior studies, we believe this approach largely eliminates the possibility that the lower disc height in HA-Young rats existed prior to HA exposure. This supports the conclusion that the observed differences reflect the effects of HA exposure rather than baseline imbalances or other potential confounding factors. The low-pressure, low-oxygen environment cannot fully simulate the unique characteristics of HA environments, but low-pressure, low-oxygen is the most important factor affecting physiology in HA environments. We placed the rats in an HA environment for 4 weeks, which cannot fully mimic the physiopathologic changes that occur in individuals living at high altitudes for a long period. In addition, placing rats at an altitude of 5800 m for a long period seems to be slightly extreme, and the rats we cultured were very prone to death in this environment, which could provide some insights for future researchers studying this model. The aging model at high altitude is not rigorous because the rats were not cultured from 5 months of age to 18 months of age in an HA environment. Finally, our study is at the transcriptome level, and the reliability of the relevant conclusions needs further experimental verification, which is one of the future works we will carry out.

In conclusion, the results of this study clarify for the first time that a low-pressure, low-oxygen-simulated HA environment aggravates disc degeneration, and this model may provide reference value for subsequent transcriptome-based analyses of the effects of HA conditions on caudal disc degeneration in rats at different ages. Enrichment analysis suggested that the effect of high altitude on IDD may result from senescence-related cycle arrest and that the regulatory effect on the cell cycle may occur through the PI3K/AKT signaling pathway.

## Conclusion

Our findings clarify the effect of high altitude on disc degeneration and predict potential molecular targets that can be used to identify or intervene in disc degeneration caused by high altitude by mining the common features of NP cells associated with the aging process in HA environments through transcriptome sequencing.

## Data Availability

Transcriptome sequencing data is available in the NCBI repository with the accession number: PRJNA1358599. Additional experimental data may be obtained upon reasonable request by contacting the corresponding author.
