# Supplementary material for: Effects of high-altitude environments on intervertebral disc degeneration and transcriptome profiling of the nucleus pulposus
Source: Front Cell Dev Biol. 2025 Dec 12;13:1709844. doi: 10.3389/fcell.2025.1709844 (PMC12741081; doi:10.3389/fcell.2025.1709844)
Supplement: Supplementary file 1 [file Table1.docx]

**Tables**

**Table 1.** Primary antibodies

| Primary antibody | Manufacturer | Catalog  number |
| --- | --- | --- |
| ACAN | PTG | 13880-1-AP |
| COL2A1 | Arigobio | ARG20787 |
| MMP3 | Abways | CY5188 |
| SFN(14-3-3σ) | Aladdin | Ab086611 |
| CS | Abcam | Ab11570 |
| COL1A1 | Santa | SC-52658 |
| P16 | Invitrogen | MA5-17142 |
