# Supplementary material for: Effects of high-altitude environments on intervertebral disc degeneration and transcriptome profiling of the nucleus pulposus
Source: Front Cell Dev Biol. 2025 Dec 12;13:1709844. doi: 10.3389/fcell.2025.1709844 (PMC12741081; doi:10.3389/fcell.2025.1709844)
Supplement: Supplementary file 2 [file DataSheet1.docx]

**Supplementary Materials**

This file includes:

Supplementary Figures 1 to 4


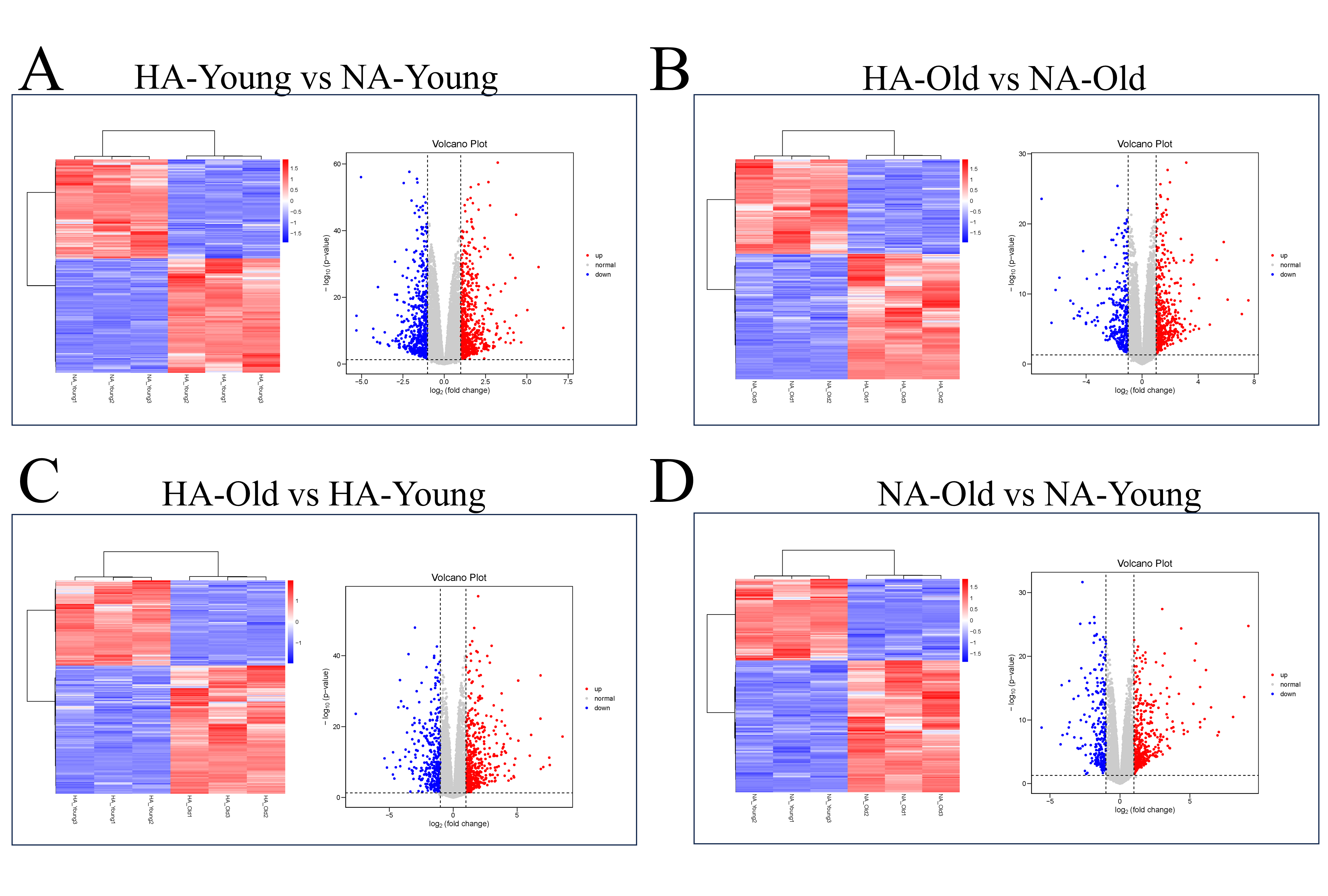


**Supplementary Fig. 1(Fig S1).** (A–D) Heatmaps of differences and clustering of expressed genes in different comparison groups and volcano maps.


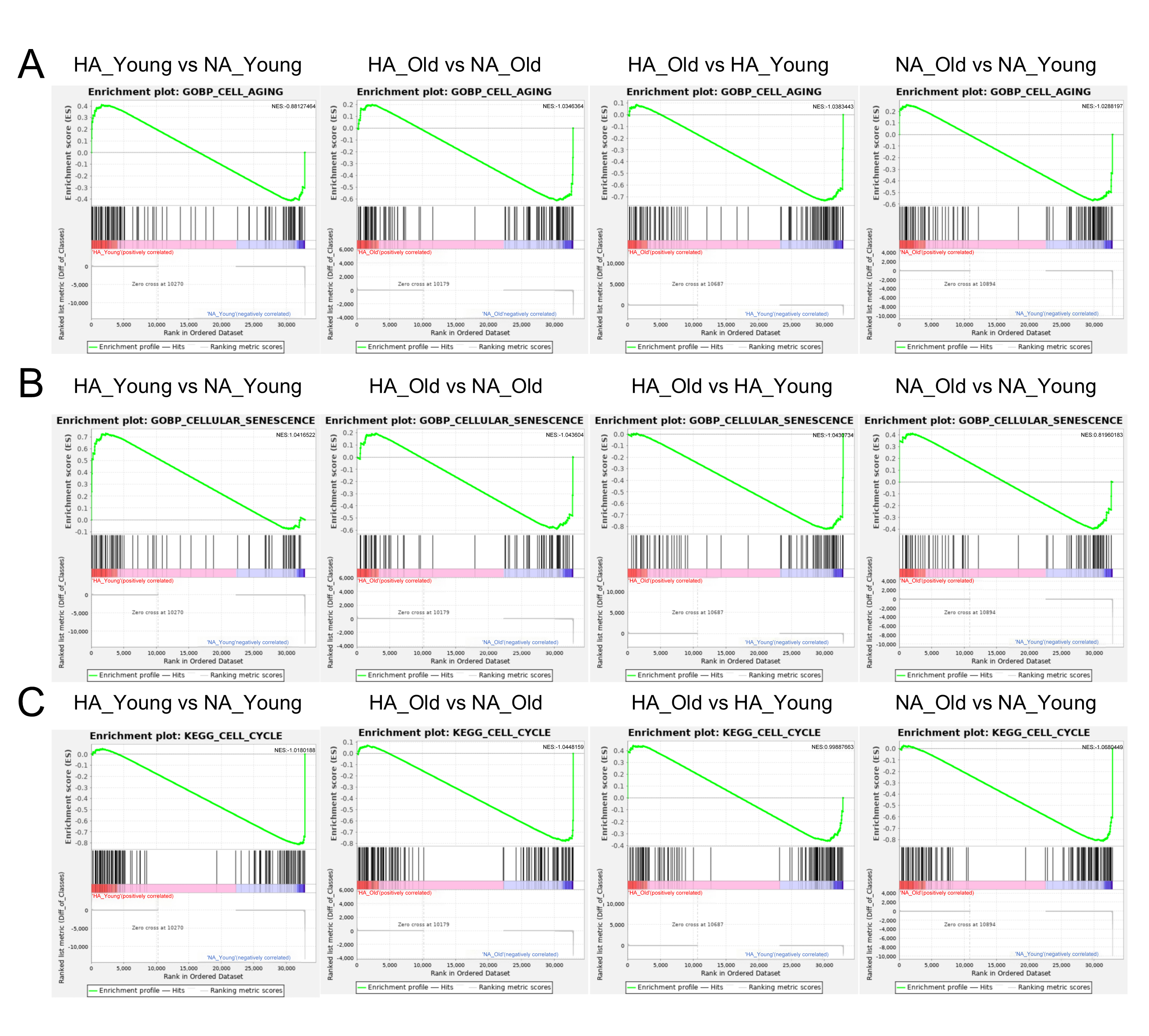


**Supplementary Fig. 2(Fig S2).** GSEA of DEGs. (A-C) Changes in the cell aging gene set, cellular senescence gene set, and cell cycle gene set in HA-YOUNG vs. NA-YOUNG, HA-OLD vs. NA-OLD, HA-OLD vs. HA-YOUNG, and NA-OLD vs. NA-YOUNG. HA-YOUNG = 5-month-old rats at high altitude (5800 m); NA-YOUNG = 5-month-old rats at nonhigh altitude (200 m); HA-Old = 18-month-old rats at high altitude (5800 m); NA-Old = 5-month-old rats at high altitude (200 m).


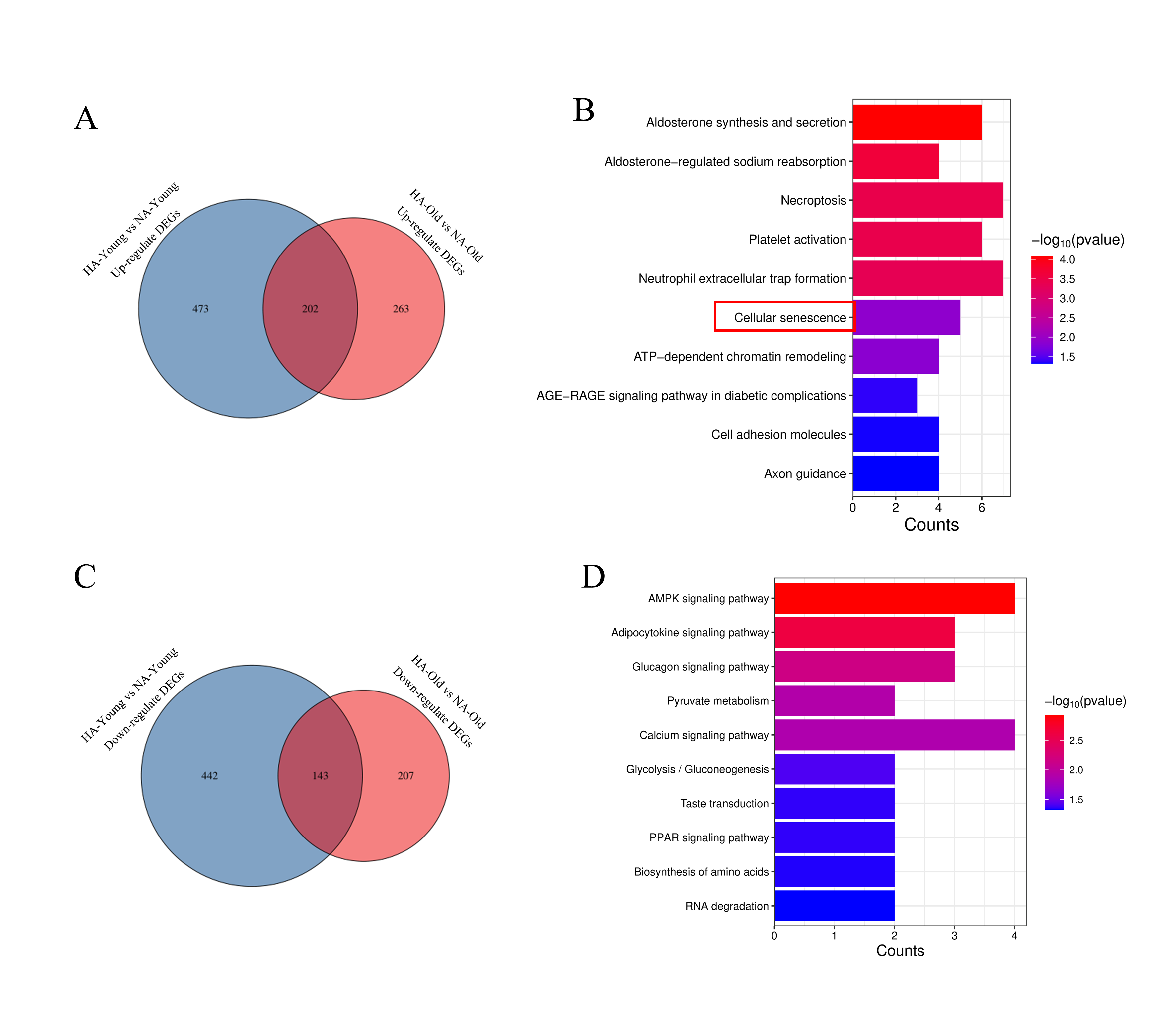


**Supplementary Fig. 3(Fig S3).** Common underlying mechanism of IDD in rats of different ages due to high altitude. (A) Coupregulated genes in HA-YOUNG vs. NA-YOUNG and HA-OLD vs. NA-OLD. (B) KEGG pathway analysis of coupregulated genes. (C) Codownregulated genes in HA-YOUNG vs. NA-YOUNG and HA-OLD vs. NA-OLD. (D) KEGG pathway analysis of codownregulated genes.


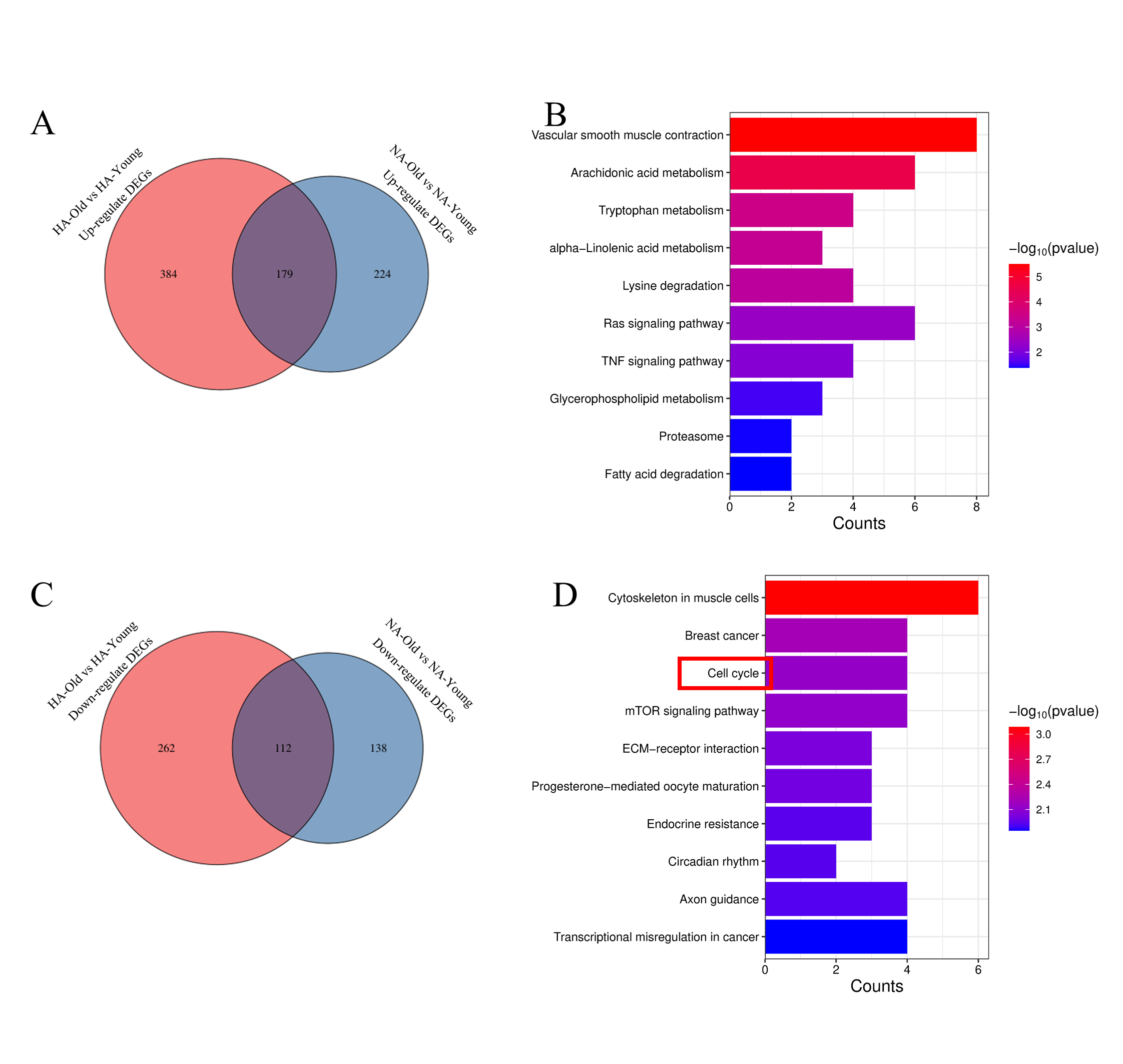


**Supplementary Fig. 4(Fig S4).** Common underlying mechanisms of age-related IDD in rats at different altitudes. (A) Coupregulated genes in HA-OLD vs. HA-YOUNG and NA-OLD vs. NA-YOUNG. (B) KEGG pathway analysis of coupregulated genes. (C) Codownregulated genes in HA-OLD vs. HA-YOUNG and NA-OLD vs. NA-YOUNG. (D) KEGG pathway analysis of codownregulated genes.
